# Corneal deflection amplitude and visual field progression in primary open-angle glaucoma

**DOI:** 10.1371/journal.pone.0220655

**Published:** 2019-08-12

**Authors:** Younhea Jung, Heejeong Chun, Jung Il Moon

**Affiliations:** Department of Ophthalmology, College of Medicine, Yeouido St. Mary’s Hospital, The Catholic University of Korea, Seoul, Republic of Korea; IRCCS Fondazione G.B.Bietti, ITALY

## Abstract

**Purpose:**

To investigate the relationship between corneal deflection amplitude and visual field progression rate in patients with primary open-angle glaucoma (POAG).

**Methods:**

This study included 113 eyes of 65 patients with POAG followed for an average of 4.81 ± 1.24 years. Evaluation of visual field progression rate was performed using mean deviation of standard automated perimetry. Corneal deflection amplitude was measured using Corvis ST (Oculus Optikgeräte GmbH, Wetzlar, Germany). Linear mixed models were performed to determine the relationship between corneal deflection amplitude, intraocular pressure (IOP), and visual field progression rate.

**Results:**

Mean age was 56.36 ± 14.58 years. Baseline average mean deviation was -8.20 ± 9.12 dB and mean treated IOP was 14.38 ± 3.08 mmHg. Average deflection amplitude was 0.90 ± 0.13 mm. In both univariate and multivariate analysis, IOP (*P* = 0.028 and P < 0.001, respectively) and deflection amplitude (*P* = 0.034 and P < 0.001, respectively) significantly affected visual field progression rate. Eyes with high IOP and greater deflection amplitude showed faster progression rate.

**Conclusions:**

Corneal deflection amplitude was significantly related with glaucoma progression. Eyes with greater corneal deflection amplitude showed faster visual field progression rate in patients with POAG.

## Introduction

While glaucoma is a progressive disease, the rate of progression varies widely among patients.[1, 2] Therefore, identifying risk factors related with progression rate may aid clinicians identify patients at high risk for fast progression. Numerous studies have reported high intraocular pressure (IOP) as main risk factor for glaucoma progression.[3–6] In addition, thin central corneal thickness (CCT) has also been identified as risk factor for progression of the disease in the Early Manifest Glaucoma Trial.[5] This could be due to underestimation of IOP in those with thinner CCT, but it may also be speculated that thinner CCT reflect the anatomical structure of the ocular tissues. In line with this speculation, corneal hysteresis has also been suggested as risk factor for glaucoma progression in several studies.[7–12] The biomechanical property of the cornea might reflect the elasticity and distensibility of the posterior ocular tissues leading to increased susceptibility of the optic nerve head to glaucomatous damage.

More recently, Corvis ST (Oculus Optikgeräte GmbH, Wetzlar, Germany) was introduced which measures the corneal deformation process in response to an air impulse and allows *in vivo* imaging of corneal biomechanical responses.[13–17] It has been suggested to measure the changes in the elastic properties of the cornea, such as how stiff or soft the cornea is, and has been reported to be more useful in detecting the true biomechanical property of the cornea than ORA.[18]

The purpose of this study was to investigate the relationship between corneal biomechanical property measured by Corvis ST, deflection amplitude, and visual field progression rate in glaucoma patients.

## Materials and methods

The study was approved by the Institutional Review Board (IRB) of Yeouido St. Mary’s Hospital (SC18RESI0149) and adhered to the tenets of the Declaration of Helsinki. In this retrospective study, informed consent was waived by the IRB, because the data were analyzed anonymously. The medical records of patients who visited the glaucoma clinic at Yeouido St. Mary’s Hospital, College of Medicine, The Catholic University of Korea, with established glaucoma between June and July 2018 were reviewed. Glaucoma was defined as open angle on gonioscopy, a normal anterior chamber based on slit-lamp examination, a glaucomatous optic disc (localized or diffuse neuroretinal rim loss, excavation, or retinal nerve fiber layer defects), and an abnormal visual field consistent with glaucoma (<20% of fixation loss, <15% of false-positive error, and <15% of false-negative error) on at least two consecutive tests. We excluded the first visual field examination from the analyses to reduce the influence of learning effects. After exclusion, those with at least 3 reliable standard automated perimetry (SAP) tests during a minimum of 3 years of follow-up or with at least 5 reliable tests in less than 3 years were included in the study. If a subject underwent surgical or laser treatment, only data prior to the treatment was analyzed. Subjects who presented best-corrected visual acuity <20/40, spherical refraction outside ± 5 diopters or cylinder correction greater than 3 diopters, history of cornea disease, ocular trauma or surgery, or previous refractive laser treatment were excluded from the study. Those with any other ocular or neurologic disease that could influence the visual field were also excluded.

Results of ophthalmologic examination and medical history of each patient were reviewed from the clinical notes including best-corrected visual acuity, slit-lamp biomicroscopy, gonioscopy, Goldmann applanation tonometry, refraction (RK-5; Canon, Tokyo, Japan), ultrasound pachymetry (Tomey, Nagoya, Japan), dilated stereoscopic optic nerve head examination, color optic disc photography (VX-10; Kowa Optimed, Tokyo, Japan), standard automated perimetry (SAP) using the 24–2 Swedish Interactive Threshold Algorithm (Humphrey Visual Field Analyzer; Carl Zeiss Meditec, Dublin, CA), and measurement of corneal deflection amplitude with Corvis ST (software ver. 1.5r1902, Oculus Optikgeräte GmbH, Wetzlar, Germany).

### Corvis ST measurements

The corneal deflection amplitude was measured with Corvis ST. Details of the Corvis ST measurements have been described previously[16]. In brief, after centering the patient’s cornea at an 11 mm-distance from the device, an air-puff at a pressure of 25 kPa was automatically emitted. During the process, an ultra-high-speed Scheimpflug camera imaged 140 digital frames of the response of the central 8.5 mm of the cornea with a resolution of 640x480 pixels over 30 msec. The cornea moved inward until it reaches the highest concavity in response to the air puff. At this time, the amount of corneal displacement at highest concavity, the deformation amplitude, is measured by the device. Deformation amplitude is composed of the deflection amplitude, which is the pure corneal component, and the whole-eye movement, which is the orbital component.[19–21] The deflection amplitude was used for the analysis.

### Statistical analyses

The rate of visual field deterioration was calculated retrospectively using the slope of the mean deviation. Linear mixed model was constructed to predict the deterioration rate using age, spherical equivalent, mean treated IOP, central corneal thickness, and deflection amplitude. In addition, we explored the interaction between IOP and deflection amplitude. Statistical analyses were performed using the SPSS (ver. 17.0; SPSS Inc, Chicago, IL). A *P* value < 0.05 was used to indicate statistical significance in all analyses.

## Results

**Fig 1:**
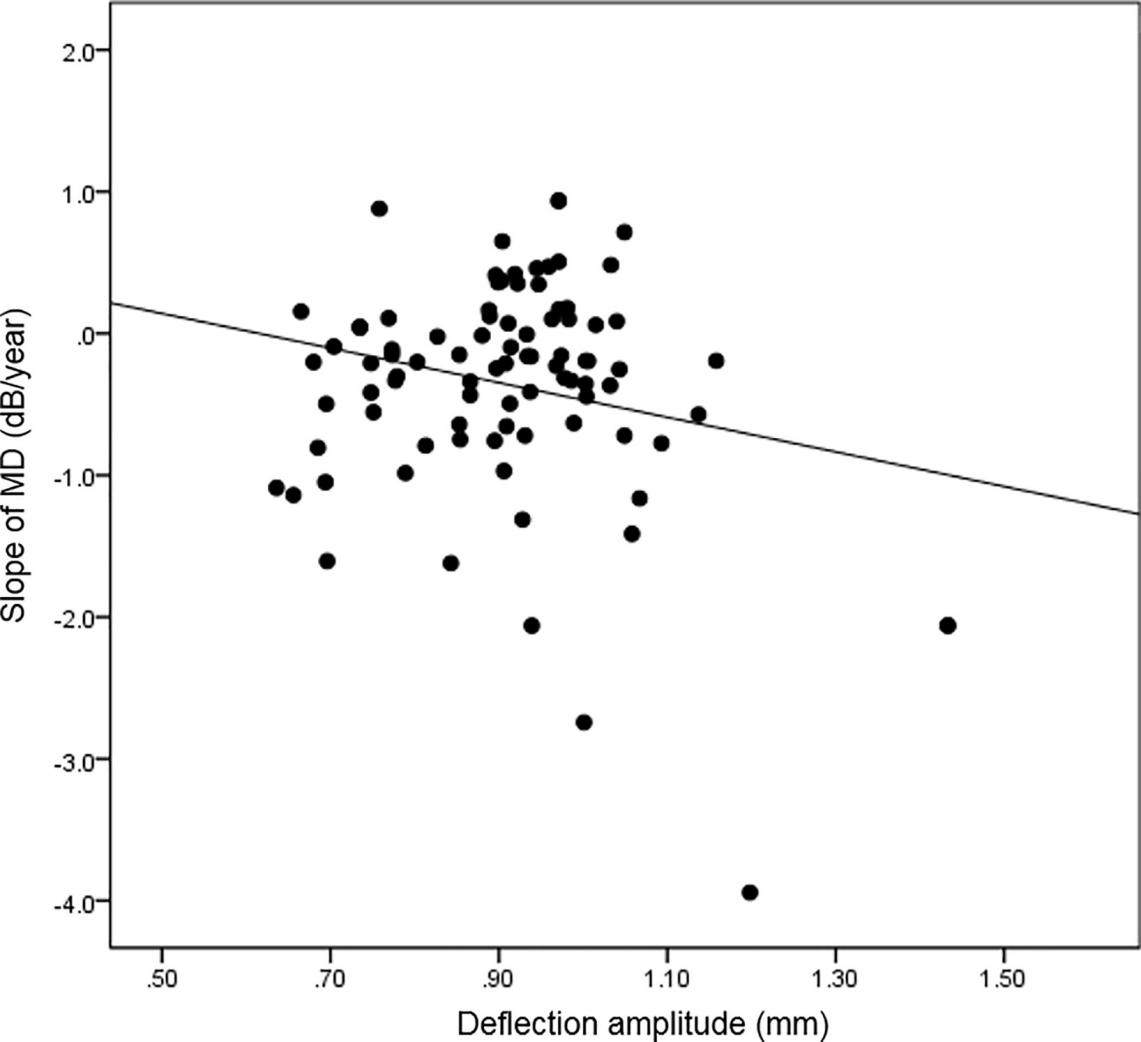
Scatter plot showing the relationship between corneal deflection amplitude and visual field progression rate. MD = mean deviation.

**Fig 2:**
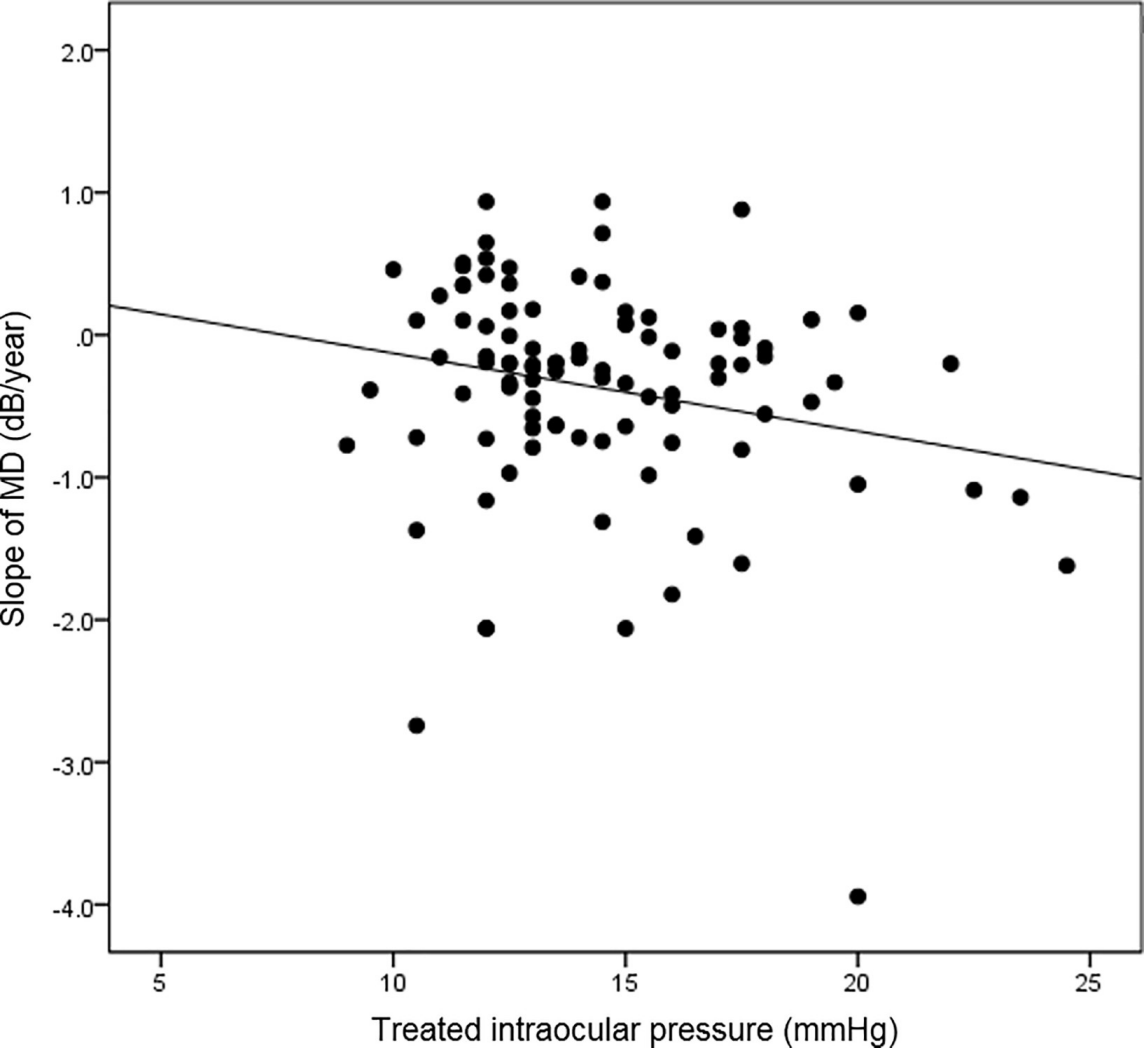
Scatter plot showing the relationship between treated intraocular pressure and visual field progression rate. MD = mean deviation.

**Fig 3:**
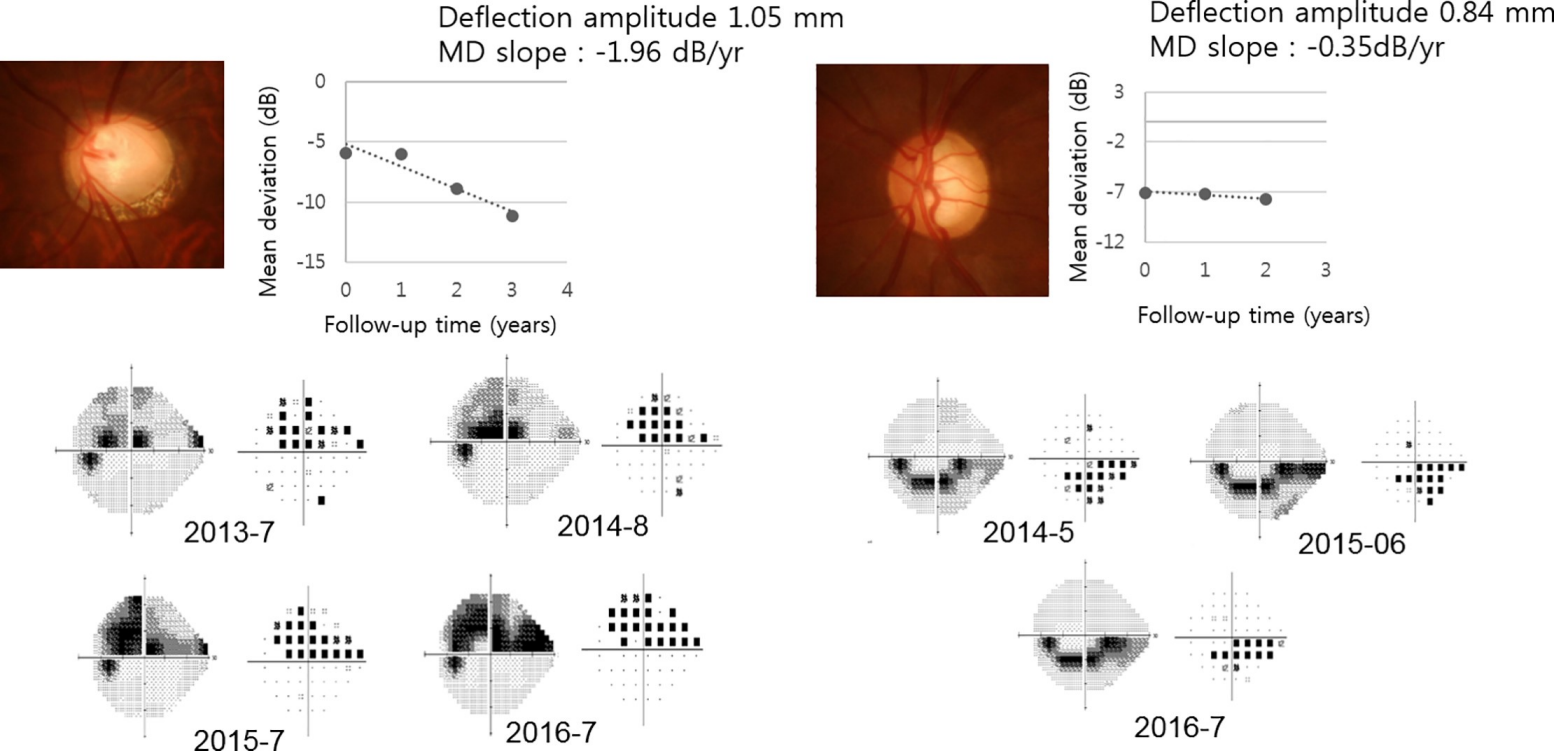
Representative cases. Progression rate (-1.96 dB/yr, B) of patient 1 (A-D) with higher deflection amplitude (1.05mm, D) was greater than that (-0.35 dB/yr, F) of patient 2 (E-H) whose deflection amplitude was smaller (0.84mm, H). S1 and S2 Videos show the whole corneal responses of Fig 3D and 3H, respectively.

A total of 113 eyes from 65 patients with glaucoma were included in the current study (Table 1). The mean age was 56. 36 ± 14.58 years and 32 subjects were male. The mean treated intraocular pressure was 14.38 ± 3.08 mmHg and mean central corneal thickness was 532.72 ± 37.08 ㎛. The mean follow-up period was 4.81 ± 1.24 years. Baseline mean deviation and visual field index were -8.20 ± 9.12 decibels and 79.64 ± 28.77, respectively. The mean corneal deflection amplitude was 0.90 ± 0.13 mm. In the univariate linear mixed model, higher intraocular pressure (unstandardized coefficient = -0.055, standardized coefficient = -0.226, P value = 0.028) and higher deflection amplitude (unstandardized coefficient = -1.220, standardized coefficient = -0.219, P value = 0.034) were significantly associated with faster rate of visual field progression (Table 2). In addition, in the multivariate model, higher intraocular pressure (unstandardized coefficient = -0.155, standardized coefficient = -0.614, P value < 0.001) and higher deflection amplitude (unstandardized coefficient = -3.370, standardized coefficient = -0.415, P value < 0.001) were both significantly associated with faster rate of visual field progression. Figs 1 and 2 show the relationships between visual field progression rate and deflection amplitude and intraocular pressure, respectively. We also explored the interaction between IOP and deflection amplitude, which showed no statistical significance (P value = 0.789). Fig 3 shows the representative cases, and S1 and S2 Videos show the whole corneal responses of Fig 3D and 3H, respectively.

**Table 1:** Clinical and demographic characteristics.

N = 65 (113 eyes)	Mean ± standard deviation
Age (years)	56.36 ± 14.58
Gender, male/female	32 (58 eyes)/33 (55 eyes)
Spherical equivalent (D)	-2.18 ± 3.15
Keratometry (D)	43.93 ± 1.71
Intraocular pressure (mmHg)	14.38 ± 3.08
Central corneal thickness (㎛)	532.72 ± 37.08
Number of visual fields	6.82 ± 2.49
Rate of visual field progression (dB/year)	-0.35 ± 0.73
Follow up (years)	4.81 ± 1.24
Baseline mean deviation (dB)	-8.20 ± 9.12
Baseline visual field index	79.64 ± 28.77
Baseline RNFL thickness (㎛)	75.32 ± 14.29
Deflection amplitude (mm)	0.90 ± 0.13

RNFL: retinal nerve fiber layer

**Table 2:** Results of linear mixed effects model investigating the effect of deflection amplitude on visual field progression rate.

Variable	Univariate				Multivariate			
	B	β	R2	*p*	B	β	*p*	Interaction *p*^†^
Age (years)	-0.010	-0.184	0.034	0.065				
Spherical equivalent (D)	0.017	0.068	0.005	0.517				
Central corneal thickness (μm)	0.002	0.112	0.012	0.263				
IOP (mmHg)	-0.055	-0.226	0.051	0.028*	-0.155	-0.614	<0.001*	0.789
Deflection amplitude (mm)	-1.220	-0.219	0.048	0.034*	-3.370	-0.415	<0.001*	

B: unstandardized coefficient

β: standardized coefficient

## Discussion

In this study, we showed that higher deflection amplitude was significantly related with faster progression of visual field in glaucoma patients. This association was significant even after adjusting for IOP. To the best of our knowledge, this is the first study to report the corneal deflection amplitude as a risk factor for glaucoma progression.

While it is difficult to clarify whether the large corneal deflection amplitude was a result of or a causative factor of visual field progression because we did not measure the corneal deflection amplitude at baseline, there can be several speculations for our findings. First, the intraocular pressure of those with high corneal deflection amplitude may have been underestimated, indicating they may have actually had higher intraocular pressure resulting in faster deterioration. Second, those who showed faster visual field progression may have been treated more vigorously, resulting in changes in corneal biomechanics. Long-term use of antiglaucoma eyedrops have been reported to have an effect on the biomechanical properties of the cornea.[22–25] Wu et al.[25] reported that chronic use of prostaglandin analogues was related with significantly larger deformation amplitude compared to naïve glaucomatous eyes. Other studies have also shown changes in corneal biomechanical properties after using topical prostaglandin analogues.[22, 24] Prostaglandin analogues can increase matrix metalloproteinase levels and remodel extracellular matrix resulting in altered corneal biomechanics.[26, 27]

More importantly, as the biomechanical properties of the cornea reflect the extracellular matrix compositions of the cornea, which could be related to those of the lamina cribrosa and peripapillary sclera, high corneal deflection amplitude may be a marker of increased susceptibility of optic disc to glaucomatous damage. We have previously reported that corneal deformation amplitude was associated with peripapillary atrophy area in patients with glaucoma.[16] The biomechanical properties of these load-bearing structures determine how they are deformed in response to IOP-related stress.[28] Stiffer eyes were less prone to biomechanical changes induced by chronic IOP elevation in experimental models, which is compatible to our findings.[29, 30] Steinhart et al. [30] reported that mice with stiffer sclera showed less loss of retinal ganglion cells in response to IOP elevation and speculated that scleral stiffening in glaucoma may protect the optic disc by increased load carried in the sclera.

The relationship between corneal biomechanical property and glaucoma progression has been previously reported using another instrument, the Ocular Response Analyzer (ORA).[8–10] Medeiros et al. [8] reported that lower corneal hysteresis was related with faster rate of visual field loss, suggesting that corneal hysteresis is a risk factor to glaucoma progression. However, corneal hysteresis measured by ORA and corneal deflection amplitude measured by Corvis ST measure different biomechanical properties of the cornea. Hysteresis of viscoelastic materials is a measure of the energy absorption during the loading-unloading, stress-strain cycle and the amount of energy absorption is calculated as the area surrounded by the loading-unloading curves[31,32]; whereas greater deflection amplitude refers to the change of shape of the loading-unloading curves.[32] Previous study has shown that Corvis ST may be more useful in measuring the true biomechanical property of the cornea.[18]

In conclusion, higher corneal deflection amplitude was significantly related with faster visual field progression rate in patients with glaucoma, and more aggressive intraocular pressure reduction may be indicated in these patients.

## Supporting information

S1 Dataset Minimal data set. (XLS)

S1 Video The whole corneal response video of patient 1 with higher deflection amplitude (1.05mm, Fig 3D) and faster rate of progression (-1.96 dB/yr). (AVI)

S2 Video The whole corneal response video of patient 2 with smaller deflection amplitude (0.84mm, Fig 3H) and slower rate of progression (-0.35 dB/yr). (AVI)
